# Online Safety When Considering Self-Harm and Suicide-Related Content: Qualitative Focus Group Study With Young People, Policy Makers, and Social Media Industry Professionals

**DOI:** 10.2196/66321

**Published:** 2025-03-10

**Authors:** Louise La Sala, Amanda Sabo, Maria Michail, Pinar Thorn, Michelle Lamblin, Vivienne Browne, Jo Robinson

**Affiliations:** 1 Orygen Parkville Australia; 2 Centre for Youth Mental Health University of Melbourne Melbourne Australia; 3 Institute for Mental Health, School of Psychology University of Birmingham Birmingham United Kingdom

**Keywords:** young people, suicide prevention, self-harm, social media, online safety, policy

## Abstract

**Background:**

Young people are disproportionately impacted by self-harm and suicide, and concerns exist regarding the role of social media and exposure to unsafe content. Governments and social media companies have taken various approaches to address online safety for young people when it comes to self-harm and suicide; however, little is known about whether key stakeholders believe current approaches are fit-for-purpose.

**Objective:**

From the perspective of young people, policy makers and professionals who work within the social media industry, this study aimed to explore (1) the perceived challenges and views regarding young people communicating on social media about self-harm and suicide, and (2) what more social media companies and governments could be doing to address these issues and keep young people safe online.

**Methods:**

This qualitative study involved 6 focus groups with Australian young people aged 12-25 years (n=7), Australian policy makers (n=14), and professionals from the global social media industry (n=7). Framework analysis was used to summarize and chart the data for each stakeholder group.

**Results:**

In total, 3 primary themes and six subthemes are presented: (1) challenges and concerns, including the reasons for, and challenges related to, online communication about self-harm and suicide as well as reasoning with a deterministic narrative of harm; (2) roles and responsibilities regarding online safety and suicide prevention, including who is responsible and where responsibility starts and stops, as well as the need for better collaborations; and (3) future approaches and potential solutions, acknowledging the limitations of current safety tools and policies, and calling for innovation and new ideas.

**Conclusions:**

Our findings highlight tensions surrounding roles and responsibilities in ensuring youth online safety and offer perspectives on how social media companies can support young people discussing self-harm and suicide online. They also support the importance of cross-industry collaborations and consideration of social media in future suicide prevention solutions intended to support young people.

## Introduction

Suicide is a leading cause of youth mortality, and in many countries, including Australia, rates appear to be increasing [1,2]. Self-harm is more common and presents a significant risk factor for suicide [3]. The reasons for self-harm and suicide are complex, with many questioning the role of social media. Concerns exist regarding the potential for certain types of online content, such as graphic depictions of self-harm or suicide methods, livestreams of suicidal behavior, or online suicide pacts or challenges to cause distress and contribute to imitative behavior [4-9]. Recent research suggests as many as 83% of young people have seen self-harm or suicide content on social media, often without searching for it, and often before the age of 14 [10,11]. Given the ways that social media algorithms operate, this may lead to young people being overexposed to potentially harmful content when interacting with their peers online [12,13].

Despite this potential for harm, young people have identified numerous benefits of using social media to communicate about self-harm and suicide. For example, it allows them to cultivate community, validate their experiences, support those they care about, and grieve people who have died by suicide [14,15]. Given the difficulties accessing high-quality, timely, and age-appropriate mental health support, young people have also identified social media as an accessible, and sometimes preferred, avenue for seeking help or information, especially for stigmatized topics such as self-harm and suicide [16]. Recognizing the central role of social media in young people’s lives, recent initiatives have capitalized on the ability to deliver highly personalized and safe suicide prevention content directly to individuals’ newsfeeds, including during suicide bereavement [17-21]. Such social media-based interventions present a significant change to the ways young people can seek evidence-based information and access mental health care [22-24].

The potential for exposure to harmful content has placed pressure on policy makers to keep young people safe online [25,26]. National suicide prevention strategies play an important role in shaping a comprehensive and systematic approach to suicide prevention; they also help determine research priorities and the allocation of resources [2,27]. However, online safety is largely absent in national suicide prevention strategies. Almost 40 countries (including Australia) have developed suicide prevention strategies [2], many of which identify young people as a priority population and consider the settings where they spend their time, for example, schools. While many national strategies recommend the development of guidelines for mainstream media to facilitate safe reporting about suicide [25,28,29], social media has only recently been included as an additional consideration for suicide prevention efforts [30-32].

Similarly, governments in many countries have developed rigorous online safety policies that focus on safeguarding the rights and privacy of individuals and regulating technology and social media companies (eg, The Online Safety Act in the United Kingdom [33]; The Online Safety Act in Australia [34], and The Children’s Online Privacy Protection Rule [35] and Kids Online Safety Act [36] in the United States). However, only a handful of these online safety policies include guidance on suicide prevention specifically [33,37]. In the absence of self-harm and suicide-specific guidance, many popular social media platforms have developed their own policies relating to self-harm- and suicide-related content [38-41]. While it is not clear if these policies are evidence-based, they differ across each platform, and social media companies are legislated and regulated differently in different countries. To this end, it appears that national suicide prevention strategies and online safety policies are yet to appropriately consider how self-harm and suicide are expressed, and managed, on social media, leaving a key gap in youth online safety policy and practice.

The objective of this study was to inform health, communication, and online safety policy as it relates to self-harm and suicide prevention. From the perspective of young people, policy makers and professionals who work within the social media industry, the aims of this study were to understand (1) relevant stakeholder views regarding young people communicating on social media about self-harm and suicide, and (2) what more, if anything, social media companies and governments could be doing to address these issues and keep young people safe online.

## Methods

### Study Design

This qualitative study involved focus groups with Australian young people, policy makers, and professionals from global social media companies. The study was conducted by researchers based in Melbourne, New South Wales, Australia, and has been reported in line with the Consolidated Criteria for Reporting Qualitative Research (COREQ; Multimedia Appendix 1) [42].

### Research Team and Reflexivity

Members of the research team who have been trained in qualitative research methods facilitated the focus groups (JR and LLS) and undertook data coding and analysis (LLS, JR, MM, and PT). All members of the research team identified as female and were employed in suicide prevention research (JR, PhD; LLS, PhD; AS, Hons; PT, MPsych [Clin]; MM, PhD; and ML, PhD) or youth mental health organizations (VB).

Focus group facilitators were known to some of the participants prior to their involvement in this study, based on their known roles and responsibilities within their respective organizations or prior involvement in other activities conducted by the research team. Participants were notified of the purpose of this study and how the data would also be used to inform the second edition of the #chatsafe guidelines, developed by the research team [21,43]. The facilitators made efforts to discuss the topics broadly and reduce any perceived pressure to provide desirable feedback related to the #chatsafe program of work by monitoring tone, verbal and body language, and engaging equally with the challenges and solutions about online communication about self-harm and suicide.

### Sample and Recruitment

In total, 6 focus groups were conducted: one with young people (n=7); 2 with Australian policy makers (n=14; these included policy makers in a range of positions and departments focused on young people, online safety, education, and health); and 3 with individuals employed by social media companies (n=7; these included staff from different companies that operate globally, whose business names will not be reported to protect confidentiality). Focus groups with professionals from social media companies were restricted to include only individuals employed by the same company in order to preserve privacy, maintain confidentiality, and foster open conversation. Each of these focus groups therefore involved a smaller number of participants (n=2-3). Young people were recruited via social media advertising on the #chatsafe social media pages, and young people who had previously participated in #chatsafe activities were invited via email. Policy makers and professionals from the social media companies were invited via email by JR.

Young people were eligible to participate if they were aged between 12 and 25 years; able to speak and read English, and; if under 18, had parent or guardian consent. Policy makers and professionals from social media companies were eligible to participate if they were older than 18 years and were (1) employed by a government department or had a policy-making role with responsibility for mental health, youth, or online safety, or (2) employed in an online safety or policy team within the social media industry. There were no exclusion criteria.

### Data Collection and Analysis

Focus groups were conducted between June 2022 and August 2022 by JR and LLS, with assistance from VB. In total, 3 were conducted online via Zoom, and 3 were conducted in a hybrid format (ie, some participants were present in-person, and others joined via Zoom). Focus groups ran for 60-120 minutes. A semi-structured topic guide was used (see Multimedia Appendix 2) and included questions about participants’ views regarding the challenges associated with young people using social media to communicate about self-harm and suicide and what more, if anything, social media platforms and policy makers could be doing to keep people safe online. Participants were also asked questions about Edition 2 of the #chatsafe guidelines, which have been reported elsewhere [21].

All focus groups were audio recorded and transcribed verbatim. Field notes were also taken. Data were analyzed using framework analysis, a structured process of theme-based analysis through the development of charts [44,45]. In conducting this study, a goal of the research team was to inform health, communication, and online safety policy as it relates to online safety and suicide prevention. As such, framework analysis was selected as the most appropriate methodology to systematically reduce the data whilst representing each stakeholder group as a “case” (eg, young people, social media professionals, and policy makers). A combined deductive and inductive coding approach allowed the research team to examine challenges and opportunities expected to emerge based on the previous literature and their own experience (eg, difficulties determining safe versus unsafe content [14] and the impact of exposure to graphic content [15]), whilst leaving scope for participants to assign their own meaning to the issues being explored.

Following transcription, six steps were followed: (1) familiarization, (2) coding, (3) development of an analytic framework, (4) applying the framework, (5) charting the data, and (6) interpreting the data [44,46]. Initially, 4 members of the research team (LLS, JR, MM, and PT) independently coded the same transcript line by line, highlighting interesting segments of the text and making notes. They met to discuss their approach and alignment on codes. In total, 3 members of the research team (LLS, JR, and MM) then coded 2 more transcripts, again meeting to discuss coded sections and interpretation of data. Where there were disagreements, the team revisited the transcript and discussed it as a group. The team agreed on a set of codes that formed an initial analytic framework. This included codes related to previously documented “harms” and “benefits” that were present in the data, as well as new codes that reflected sentiments raised by participants regarding solutions, challenges, and what more different stakeholders could do to keep young people safe online. LLS coded the remaining transcripts, meeting with MM and JR regularly, and iteratively revised the thematic framework used to interpret the full data. Transcripts were coded by hand, and codes, categories, and representative quotes from each case were charted in an Excel spreadsheet by LLS. The matrix was reviewed and discussed by LLS, JR, MM, and AS, and connections between and within the categories were thematically mapped as they addressed the research questions.

### Ethical Considerations

This study received ethics approval from the University of Melbourne Human Research and Ethics Committee (ID: 22728). Participants were required to complete an online consent form and demographic survey prior to attending their focus group. All data were de-identified for analysis. Youth participants were reimbursed Aus $30 (US $19.02) per hour for their involvement in this study. Policy makers and professionals working within the social media industry received no compensation.

## Results

### Participants

Participant demographics are summarized in Table 1, including information about the top 3 social media platforms used by participants in each group. Additional information about professional participants’ current employment was also collected, though some details have been omitted to avoid possible reidentification. All but 3 participants across the total sample reported using social media platforms in their daily life, with most using 3 or more platforms (n=20, 80%).

**Table 1:** Participant demographics and top social media platforms used by policy makers, social media industry professionals, and young people.

Group (n)	Age (years) mean (range)	Sex or gender (n)	Country of birth (n)	Identifying as Aboriginal (n)	Top social media platforms used (n)
Policy makers (14)	39.31^a^ (23-54)	Female or woman (10) Male or man (3)^a^	Australia (12) Other (2)^b^	Yes (1) No (13)	Facebook (10) YouTube (8) Instagram (7)
Social media companies (7)	39.14 (27-57)	Female or woman (4) Male or man (2) Non-binary (1)	Australia (1) Other (6)^b^	No (7)	YouTube (7) Facebook (5) Instagram (5)
Young people (7)	20.71 (19-23)	Female or woman (5) Male or man (2)	Australia (3) Other (4)^b^	Yes (1) No (6)	Instagram (7) Facebook (6) YouTube (6)

^a^One policy maker participant did not disclose their age or sex or gender.

^b^Other countries of birth included Hong Kong, Malaysia, Mexico, Singapore, Taiwan, the United Kingdom, and the United States.

The resulting 3 primary themes and 6 subthemes are presented below. The analytic framework used to interpret these data, with quotes from each stakeholder group, is provided in Multimedia Appendix 3.

### Challenges and Concerns

To address the first research question, participants from all 3 stakeholder groups were asked about their views regarding young people using social media to communicate about self-harm and suicide. While all stakeholder groups were encouraged to share what they felt the challenges were and the reasons for young people using social media for this purpose, each group mostly arrived at the same conclusion: there are concerns about young people engaging with, and being exposed to, self-harm and suicide-related content on social media; however, social media is not going away and may meet the needs of young people looking for a safe place to express their distress. Perspectives of each group were also largely influenced by recent public discourse relating to social media and youth mental health more generally, with groups differing in their views regarding the helpfulness of this debate.

#### Reasons for, and Challenges Related to, Young People Using Social Media to Communicate About Self-Harm and Suicide

Participants in all focus groups expressed the belief that individuals used social media to seek and provide support or share and understand personal experiences related to self-harm and suicide. Some youth participants described social media as a place to “perform” their mental health and felt that representations of self-harm and suicide on social media were heavily impacted by what their peers were posting and the feedback that they sought. Across all focus groups, there was an appreciation for the necessity of using social media to have these conversations, as participants recognized that they are difficult topics to discuss offline, and for many people, help is not available elsewhere. There was consensus across each focus group about the affordances of social media (eg, anonymity and temporality) that make these topics easier to talk about online versus face-to-face.

It's a lot easier to just put a post out there for the world to see… if people are feeling that way, they probably feel quite like they might be a burden and they don't want to go up to their friends and say, ‘I'm feeling like this’, or they don't want to go up to a mental health practitioner and tell them. So it's easier just to put the post out into the public and see who replies back.

Young person

...social media can provide…a really accessible space for young people. I mean accessible in terms of not just for young people who live in areas where there might not be access to services. But for young people who maybe don’t feel safe going to a service… it’s… 24:7, stigma-free often, they can connect with people who are in similar situations.

Policy maker

Although individuals in all participant groups understood why and how these conversations occurred on social media, each group articulated their concerns for the harms associated with online self-harm– and suicide-related content. Concerns related to the age of the user and what is developmentally appropriate to view; the virality of self-harm and suicide content and specific online challenges; the potential for social media to sensationalize, glorify, or normalize self-harm and suicide; users being exposed to harmful content, livestreams, or real-time suicidal behavior; the potential for contagion; and other online experiences that have implications for self-harm and suicide (eg, bullying, sextortion, etc).

I remember this one instance where this girl posted on her private account - and I wasn't that close with her so I really shouldn't have been there; but a photo of a razor blade...It was framed in a certain way, and she captured it like, ‘hello, my old friend’, or something. Then two days later, another girl in another circle posted essentially the same photo… she'd definitely seen it, so there was the element of contagion.

Young person

A similar theme across each focus group was uncertainty around the nuances of what constituted harm, for whom and when, and how best to respond to online harms. Social media industry professionals described this challenge as not knowing “where to draw the line” between helpful versus harmful content, and policy makers discussed the heterogeneity in user experiences and outcomes. Young people expressed their belief that more needed to be done to prevent individuals from seeing harmful content in the first place, describing teenagers as not developmentally mature enough to understand the content that they were being exposed to regularly, based on their own experiences.

Participants from the policy maker and social media industry focus groups reflected on the complexity associated with balancing freedom of online expression with the immediate removal of harmful content. Enforcing these decisions across global corporations and different legislative environments, particularly in the absence of a strong evidence base, was a challenge frequently discussed by those developing and implementing safety policies.

It has harmful stuff on it but can provide such an avenue for help seeking as well. How do you find that balance?

Policy maker

That component of freedom of expression where we also don't want to talk over the voices of people who have historically been marginalized, especially by scientific communities or by lack of access to resources or things like that. So for us… you want to enable safe communication and also how do you do that in a way that is grounded in both research evidence and global equity?

Social media industry professional

#### Reasoning With a Deterministic Narrative of Harm

Irrespective of their personal views, participants in all stakeholder groups were aware of public perceptions that social media causes harm to mental health more broadly and had different perspectives about how helpful or harmful those perceptions were, both to individuals using social media and to those developing and regulating them. In reflecting on why people believe social media is negatively impacting youth mental health, some youth participants acknowledged that social media is often perceived as not being proactive in terms of creating safe online environments but commented that there were many other factors that impacted youth mental health beyond social media.

Perspectives amongst policy makers were more varied. One participant described their firm belief that “being on social media is [a] detriment to people’s mental health.” However, the majority of policy makers instead wondered how they could better “capitalis[e] on what social media can provide” in order to support individual users while minimizing potential harms.

Participants from the social media industry expressed feeling like a “punching bag for online safety” while reflecting on there being “no research to support this conclusion [of harm]." Some social media professionals believed that this public perception stalled more sophisticated conversations about the future potential and innovation of social media from a mental health perspective.

This growing narrative that there's a causal link between social media and self-harm…really, there's no research to support this conclusion, but I think it really hinders our abilities sometimes to put out a different narrative that can feel supportive for people, and that can help drive behavior change, that can drive people towards our platforms to utilize some of the resources that we use.

Social media industry professional

### Roles and Responsibilities

A central question across all focus groups was the issue of responsibility for online safety and where the lines of responsibility started and ended for each stakeholder group. All focus groups featured discussions about how challenging these topics were, especially when online safety and themes related to self-harm and suicide overlapped. Due to this complexity, participants in all groups appeared to articulate more questions than answers. This highlighted areas where each stakeholder group felt others could be doing more and where better collaborations and models of partnership were required.

#### Who Is Responsible and Where Does Responsibility Start and Stop?

Participants raised many rhetorical questions related to the roles and responsibilities of their industry or position. For example, participants from the social media industry acknowledged that they play a fundamental role in defining socially acceptable behavior online (and offline) through the implementation of their community guidelines or standards. One participant from the social media industry stated, “we're not a medical company and we're not a healthcare company” and questioned how appropriate it was that they were the people to ultimately determine what is safe versus unsafe behavior or content for different global regions and across different cultural settings.

From a platform perspective, we’re obviously not doing clinical work, but we’re still judging what type of content is considered to be more problematic or less problematic from a conversational perspective in terms of the risk for the person, when we need to report it to different cases, what types of resources we need to provide. So, the question becomes, like, does this distinction, for instance, between passive and active suicidal ideation, does that mirror in languages that don’t have the same language structure as English or expression?

Social media industry professional

Some described their need to develop new policies “on-the-go” to keep up with the advancement of technology and user behavior, yet they often felt that there was little empirical evidence guiding decision-making and their need to move more quickly than research can. Given their reach and popularity, one participant commented that “any one direction we move the needle, even just microscopically, is going to have an impact on a huge swath of users.” Participants from this industry also expressed a desire for “more regulation” but felt that, in some cases, regulation restricted their ability to “use their proactive technologies… to save and have a considerable impact in people's lives.” To navigate these tensions, participants from the social media industry highlighted the importance of meaningful investment from policy makers or governments regarding online safety.

… we can’t keep people safe, we can only try to protect them from harm. We can’t define safe as an absolute state of being online, offline, or anywhere else. So, what we have to do is, we have to do our best to mitigate risk and their exposure to risk.

Social media industry professional

Just as a policymaker on platform, there's really a lack of evidence around a lot of the policy questions that we're struggling with in terms of where to draw the line, especially really solid research evidence and evidence that comes from contents and cultures outside of what is typically thought of as like the WEIRD western world.

Social media industry professional

Policy makers described how the regulatory landscape influences the content that should or should not be shared online but also spoke openly about the challenges they face when trying to regulate and govern international corporations and online content that is geographically boundless. While acknowledging the limitations of government regulation, policy makers reflected on the importance of cross-government approaches and their responsibility to ensure that they are supporting individuals to stay safe online by “providing information and support and guidance in a variety of settings, in a variety of forms.”

So we are interested in health, there’s the Department of Education who has an interest here, there’s the eSafety Commissioner, the Department of Comms. So, there’s lots of different government agencies that are interested, but there's also the sites themselves. I think there's a lot of different players and sometimes that can perhaps make things even more complex than we would otherwise like.

Policy maker

Lastly, it was understood that individual users play a critical role in creating safe online environments and need to have agency over their interactions. However, all groups acknowledged that younger users could not take sole responsibility for their own safety within environments created by, and often for, adults.

I don’t like to put the safety of individuals, like, all of the responsibility for the safety of individuals, on the individuals because I think that suicide and self-harm in particular has been seen as this really individual problem that’s disconnected from everything that happens in the community…

Social media industry professional

In the absence of clear roles and responsibilities, and given the complexity of these issues, there was a tendency for participants to describe their own responsibility in relation to what more other stakeholder groups need to be doing. For instance, some participants questioned the legitimacy of government investment in online safety, and others questioned what more parents or carers, and school curricula, could do. Despite a broad recognition that collaborations were needed, participants sometimes conveyed an “us versus them” mentality, either between companies within the same industry (eg, one social media platform versus another) or between stakeholder groups (eg, the social media industry versus national governments; social media platforms versus parents).

Politicians would much rather help parents say it's someone else's fault, yours, the industries, than actually say, actually you also need to step up and do something here to promote online safety.

Social media industry professional

Young people felt strongly that the government could help by providing more education and digital literacy training, particularly when they were younger and still attending school. Structured education on online safety was perceived as a way of alleviating pressure on young people to be responsible for safe online environments in ways that they can’t “handle” yet, while also providing them with knowledge that would be protective.

I think if the government has a role to play at all, it would be through education…like those special assemblies… Then, if you have young people who have been educated in this way, they can self-police - young people are really good at that, hopefully in ways that are helpful as opposed to, you're bad for posting this, this is what we can do instead. I feel like education - all of the stories that we've shared about…seeking help and not really having the resources or tools or vocabulary, and so if you find a way to educate young people…in a more structured way, then I feel that would really alleviate a huge part of the problem.

Young person

#### The Need for Better Collaborations

Participants from all groups highlighted the need for a collaborative approach across sectors to achieve meaningful change in ensuring online safety. There was a sense that there was "a shared responsibility for this issue" and "a lot of different players," but "different levels of investments" in online safety. It was also acknowledged that these tensions are felt in the absence of "better models of partnership" or collaboration, though no concrete examples were presented for how this might be achieved.

…[it’s] going to have to include a multi-pronged approach. That includes some level of, depending on age, parental, familial, government, industry; all of these different factors I think need to come together to really, I think, uniformly protect people. In the absence of that, I think it's too easy to go one way or the other. To try to put this all on young people, to try to put this on social media or the government, it leaves out so many different other parts of this puzzle that when together, you're best placed to build that front to protect users.

Social media industry professional

Promisingly, there was a desire from all participants to work together, in acknowledgment that the issues are “big, really tough challenges that we all face,” and that issues related to “data use transparency, consent, and funding” need to be addressed collectively. There was also recognition from social media industry participants of the importance of social media companies continuing to partner with “subject-matter experts” so that their safety actions and tools were evidence-informed.

### Future Approaches and Potential Solutions

#### Overview

Participants in all focus groups were asked to reflect on current and future applications of social media as a possible tool in youth self-harm and suicide prevention. Here, participants shared their perspectives and knowledge of the use of current safety tools and policies embedded within social media platforms, highlighting issues with current safety features. Participants expressed ideas for the future of online safety and suicide prevention using new technologies (eg, artificial intelligence [AI]) to detect and respond to risk, while also acknowledging issues related to user privacy and the lack of transparency regarding current uses of recommender systems.

#### Acknowledging the Limitations of Current Safety Tools and Policies

Young people and policy makers in this study were aware that some platform safety features currently exist, but there appeared to be little faith in their ability to create safer online experiences. This either came about from not trusting the platforms to act accordingly (eg, previous experiences of using a reporting tool resulting in no action and therefore discouraging a user from doing that again) or users not knowing the full range of safety tools and features that exist or how to use them. While there was an appreciation that there is no “one-size-fits-all” approach to online safety and that not all tools and functions were going to suit each user, some believed that “having some improved transparency over those reporting processes” would foster greater trust.

Personally, I don't have much faith in social media's reporting features. There have been times – like you see some really atrocious things online… and you're like, oh my gosh, I really – I can't believe I encountered that…You report it and then five days later it's like, it's been reviewed and it's been decided there was no issue here.

Young person

… sometimes things are so awful you want to report them but then there's not a specific thing that you can allocate it to … it's just awful.

Young person

#### Scope for Innovation and New Ideas

When asked what more the broader social media industry could be doing to promote online safety and make their platforms safer for younger users, particularly youth participants and policy makers spoke about their ideas for how AI and platform algorithms, or recommender systems, could be better optimized. This included using AI tools to detect risk, respond in real-time to risk or distress, and deliver services or support.

They could be doing more...but more proactively in terms of, it seems like this post has content discussing suicide, click here for more information, and there's the chatsafe guidelines, or an iteration. It would definitely boost how social media is regarded, which is as like a necessary villain.

Young person

On the other hand, participants from the social media industry spoke about the limits of current technologies, describing that “humans are really good at certain things and algorithms are really good at others.” They felt that there were misunderstandings about how algorithms work and the extent to which AI can be used ethically while also maintaining user privacy. In some instances, current regulatory frameworks prohibit the use of risk detection technologies, but better guidance and support from policy makers was perceived to allow these technologies to advance in potentially helpful ways.

When we’re talking about AI capabilities and algorithms in particular, what I’ve found is that the people who suggest those are typically, in my experience, people who don’t have a lot of experience building algorithms…

Social media industry professional

I think as a society we are rightfully pretty skeptical of AI and algorithms when it comes to health concerns, in particular, and so without laws changing pretty significantly in a lot of countries, I think that there’s a lot of hard limitations and blocks to what we can do as a company without overstepping the boundaries of legal liability to provide help to users or provide targeted, for instance, help, like, support resources and things like that.

Social media industry professional

Despite the “technology not being there just yet,” participants from the social media industry acknowledged that the reporting functions and safety tools, with refinement, could become much more user-friendly and helpful for users. Some participants were also hopeful that future innovation in online safety could involve the integration of evidence-based interventions that are promoted or delivered in-platform.

I think that my dream would be to have better help tooling available online for folks, so really having things like having a safety plan available or having a single session intervention that is culturally responsive and appropriate available for users to be able to opt into doing … that’s evidence-based. I think that there [are] steps that the industry is taking towards getting there, I think that it will be interesting to see what happens in this space within the next five to 10 years.

Social media industry professional

While some policy makers admitted to knowing very little about AI or its capabilities, they were strongly in favor of using proactive detection technologies to promote resources and reach individuals with real-time support or help. There was also acknowledgment that Australia is well positioned to be a world leader in this regard.

I definitely do think that having links and support… with the algorithm picking up [that certain] content, but also if the algorithm can pick up that kind of content, shouldn't there also be a few ways to hopefully stop that content? Rather than just leaving it up and be like, here's some links though just in case you might click on it and have a look.

Policy maker

## Discussion

### Principal Findings

This study explored views regarding young people using social media to communicate about self-harm and suicide from the perspective of young people, policy makers, and individuals who work for social media companies. It also explored participants’ beliefs about what more social media companies and governments could be doing to enhance online safety for young people engaging with, and exposed to, self-harm and suicide-related content. Our findings reflect the tensions regarding roles and responsibilities for youth online safety, as well as various perspectives on the ways social media could be used to support young people when communicating online about these topics.

Our findings support a growing body of research exploring the reasons why young people use social media to share and understand their experiences of self-harm and suicide [14,15]. Young people using social media as an informal source of support and help-seeking appears to be beneficial in circumventing barriers associated with professional mental health care (eg, stigma, cost, and long wait times) [16] and allows young people to validate their own experiences [14]. However, participants in this study also echoed concerns associated with young people using social media for this purpose (eg, potential overexposure to harmful content, contagion effects, age of users, privacy, and access to individual data) [5,7,15,47], highlighting uncertainty around when and how these risks might translate to harm for individual users, who might be most at risk and how best to respond.

Contextualized within a time where social media companies are being heavily scrutinized for the actions that they are (or are not) taking with regards to youth safety and wellbeing [6,48-50], some participants in this study felt that a deterministic narrative around social media conferring harm stalled more sophisticated and nuanced conversations needed to understand and address these risks. The focus on social media playing a significant role in rising rates of youth psychological distress, despite little to no causal findings to support this claim and often in the absence of considering other social determinants that are impacting young people [51-54], has prohibited the expansion of our understanding about how social media impacts individuals differently and who is actually most at risk. This understanding is limited further when it comes to self-harm and suicide. Though some effort has been made to address potential online harms through research (eg, the #chatsafe intervention) [17,18,20], policy [34], and industry practice (eg, platform safety centers and tools) [38-41], it is clear that more work needs to be done to understand the precise nature and mechanisms of harm in this context and to develop evidence-informed solutions and policies that carefully consider the needs of young people.

A prevailing sentiment among policy makers and social media industry professionals was that they felt ill-equipped to make online safety decisions that were both timely (per the rapid nature of this industry) and evidence-based. Given that many of the decisions that platforms need to make quickly about self-harm and suicide-related content lack empirical evidence, there is an urgent need for better relationships between researchers and social media companies, including the sharing of data (whilst maintaining user privacy) and investment in the development of evidence-informed and age-appropriate safety policies and features. Although platforms regularly publish transparency reports quantifying the volume of content they have removed in enforcing their community guidelines [55-57], there is a notable lack of transparent information detailing how decisions around safety policy development and implementation are made, by whom, and based on what evidence [49]. Though we acknowledge the tensions in social media companies navigating transparent reporting on their systems and processes while protecting their intellectual property, greater attention does need to be given to how safety policies are developed, informed, and implemented, and there is a clear need for research evidence to support best practice. This aligns with calls from the Australian eSafety commissioner for “radical transparency” from social media companies regarding their online safety approaches [49], as well as recommendations for collaborative and cross-industry efforts to reduce social media harms proposed in the US Surgeon General’s report on social media and youth mental health [48].

The theme of roles and responsibility was central across all focus group discussions regarding what more social media companies and policy makers could be doing to promote online safety. Critically, there was a lack of agreement or clarity on the nature and extent of responsibility that should be assigned to various stakeholder groups represented in this study, but also to stakeholders such as parents, educators, and health professionals more broadly. Though cross-industry partnerships, international coalitions [26,58], and the creation of subject-matter safety advisory boards [59,60] are all currently occurring in various ways, they are often happening in isolation from one another and differ across regions and platforms. They are further challenged by differing online safety policies and capacity for regulation globally. The inclusion of online safety in future suicide prevention policies, both at a national level and by global health organizations (eg, the World Health Organization), would go some way toward clarifying the roles and responsibilities of relevant stakeholder groups and would facilitate the sharing of information and evidence-based practice. Consideration of digital contexts in suicide prevention policies would also support meaningful investment in research and the establishment of cross-industry partnerships to promote online safety for young people.

Finally, several opportunities for innovative and novel ways of using social media to detect, respond to, and manage self-harm and suicide risk were explored. These mostly related to using social media algorithms to promote helpful resources to someone identified as at-risk, with the potential for platforms to direct individuals to professional support or deliver help within the platform itself [11,61]. However, while young people and policy makers supported these ideas, platform professionals emphasized the limitations of current technologies and the misconceptions community members hold about their capacity. Further, recommender systems and algorithms are relatively new technologies that have been delivered to a huge global user base as a means of optimizing user experience and keeping individuals active on social media platforms. There are understandable questions about the ethics of their use and consideration for user privacy, as well as concerns regarding the adverse impacts of recommender systems in promoting harmful content [49]. There is also limited transparency around how these systems are developed, updated, or regulated and for how these issues may be addressed. However, platform algorithms present an important and unique opportunity for reaching young people with high-quality and evidence-informed mental health supports, especially as these systems become more advanced and personalized over time.

### Strengths and Limitations

A strength of this study is the representation of views from young people, policy makers and professionals from the social media industry; groups that are usually working in isolation from one another and fundamentally opposing one another. To the best of our knowledge, this is the first study to include the perspectives of professionals working for social media companies in discussions about youth online safety regarding self-harm and suicide. Although focus groups with these participants were smaller in size than those with the other participant groups, the decision to restrict industry focus groups to individuals employed by the same company preserved participants’ privacy and promoted open discussion within these smaller groups. Conducting a framework analysis allowed for these distinct stakeholder groups to be equally involved in this work, resulting in a more nuanced discussion of the digital landscape that young people communicate within, processes to develop and regulate those environments, and the best ways of supporting individuals in the future.

Prior knowledge of the research team by some participants was a limitation of this study. However, it did not appear that these established relationships hindered the conversations or information shared. Instead, this pre-established trust and rapport appeared to facilitate honest conversations about the challenges faced by each group. A mutual respect for the complexity and challenges associated with youth self-harm and suicide prevention also invited open conversations with a shared understanding of wanting to do more.

Finally, the average age of the youth group was approximately 21, and given that most social media platforms allow users to create accounts from as young as 13, this work would have been strengthened by the inclusion of younger participants. Further, given their prior involvement in other activities, youth participants were very mental health literate and aware of the importance of safe communication about self-harm and suicide, which may not be representative of the wider Australian youth demographic. Additional stakeholder groups, such as parents, carers, and schools, were not included in this study, though their perspectives have been reported elsewhere [62] or are being collected through work that is currently underway within our team.

### Conclusions

There are valid concerns about young people using social media to discuss self-harm and suicide. However, social media remains a preferred and accessible way for young people to communicate and seek help. The evidence base for current platform strategies to manage self-harm and suicide content is lacking, raising questions about the appropriateness of social media companies defining online safety alone, as well as the challenges governments face when trying to regulate global companies and fast-spreading content. Our findings highlight the complexity of these issues and the need for shared responsibility and greater understanding of roles across relevant stakeholder groups. These findings present strong support from young people, social media companies, and Australian policy makers for cross-industry partnerships to create safer online experiences for young people and the implementation of new and emerging technologies to prevent youth self-harm and suicide.

## Appendix

Multimedia Appendix 1 Consolidated criteria for reporting qualitative studies (COREQ) - 32-item checklist.

Multimedia Appendix 2 Semistructured topic guide used to conduct the focus groups.

Multimedia Appendix 3 Representation of thematic framework, including description of themes and subthemes, and illustrative quotes from each case.

## Abbreviations

AI: artificial intelligence
COREQ: Consolidated Criteria for Reporting Qualitative Research

## Data Availability

The datasets generated or analyzed during the current study are not publicly available due to inability to sufficiently anonymize transcripts, but are available from the corresponding author on reasonable request.
