# Supplementary material for: Online Safety When Considering Self-Harm and Suicide-Related Content: Qualitative Focus Group Study With Young People, Policy Makers, and Social Media Industry Professionals
Source: J Med Internet Res. 2025 Mar 10;27:e66321. doi: 10.2196/66321 (PMC11933773; doi:10.2196/66321)
Supplement: Multimedia Appendix 1 [file jmir_v27i1e66321_app1.pdf]

Consolidated criteria for reporting qualitative studies (COREQ): 32-item checklist

No	Item	Guide questions and *response*
**Domain 1: Research team and reflexivity**		
Personal Characteristics		
1.	Interviewer/facilitator	Which author/s conducted the interview or focus group? *Details provided in research team and reflexivity section.*
2.	Credentials	What were the researcher's credentials? *Details provided on the Title page.*
3.	Occupation	What was their occupation at the time of the study? *Details provided on the Title page.*
4.	Gender	Was the researcher male or female? *Details provided in research team and reflexivity section.*
5.	Experience and training	What experience or training did the researcher have? *Details provided in research team and reflexivity section.*
Relationship with participants		
6.	Relationship established	Was a relationship established prior to study commencement? *Details provided in research team and reflexivity section.*
7.	Participant knowledge of the interviewer	What did the participants know about the researcher? *Details provided in research team and reflexivity section.*
8.	Interviewer characteristics	What characteristics were reported about the interviewer/facilitator? e.g. Bias, assumptions,

No	Item	Guide questions and *response*
		reasons and interests in the research topic. *Details provided in research team and reflexivity section.*
**Domain 2: study design**		
Theoretical framework		
9.	Methodological orientation and Theory	What methodological orientation was stated to underpin the study? *Framework analysis is discussed as the analytic approach in the data collection and analysis section.*
Participant selection		
10.	Sampling	How were participants selected? *Sampling approach is discussed in the sample and recruitment section.*
11.	Method of approach	How were participants approached? *Method of approach for all participant groups is discussed in the sample and recruitment section.*
12.	Sample size	How many participants were in the study? *Sample size is discussed in the sample and recruitment section.*
13.	Non-participation	How many people refused to participate or dropped out? Reasons? *N/A*
Setting		
14.	Setting of data collection	Where was the data collected? e.g. *home, clinic, workplace. This is reported in the Data collection and analysis section.*

No	Item	Guide questions and *response*
15.	Presence of non-participants	Was anyone else present besides the participants and researchers? *No*
16.	Description of sample	What are the important characteristics of the sample? *Relevant participant characteristics are presented in the sample and recruitment section, as well as the results section.*
Data collection		
17.	Interview guide	Were questions, prompts, guides provided by the authors? Was it pilot tested? *Details of the interview guide are in Multimedia Appendix 2.*
18.	Repeat interviews	Were repeat interviews carried out? If yes, how many? *N/A*
19.	Audio/visual recording	Did the research use audio or visual recording to collect the data? *Audio recording is described in the data collection and analysis section.*
20.	Field notes	Were field notes made during and/or after the interview or focus group? *Yes, this is included in the data collection and analysis section.*
21.	Duration	What was the duration of the interviews or focus group? *This is included in the data collection and analysis section.*
22.	Data saturation	Was data saturation discussed? *Data saturation wasn't considered an appropriate measure of data quality for the present study. Specificity of the participant sample relative to the research aims was deemed to support adequate information power instead.*
23.	Transcripts returned	Were transcripts returned to participants for comment and/or correction? *No*

No	Item	Guide questions and *response*
**Domain 3: analysis and findings**		
Data analysis		
24.	Number of data coders	How many data coders coded the data? *Details of coding approach are discussed in the data collection and analysis section.*
25.	Description of the coding tree	Did authors provide a description of the coding tree? *Details of coding approach are discussed in the data collection and analysis section.*
26.	Derivation of themes	Were themes identified in advance or derived from the data? *Inductive and deductive coding approaches are discussed in the data collection and analysis section.*
27.	Software	What software, if applicable, was used to manage the data? *Details of coding approach are discussed in the data collection and analysis section.*
28.	Participant checking	Did participants provide feedback on the findings? *No*
Reporting		
29.	Quotations presented	Were participant quotations presented to illustrate the themes / findings? Was each quotation identified? e.g. participant number. *Yes, illustrative quotes are provided throughout the results section and are attributed based on the participant's group. Also see Multimedia Appendix 3.*
30.	Data and findings consistent	Was there consistency between the data presented and the findings? *See Results section.*

No	Item	Guide questions and *response*
31.	Clarity of major themes	Were major themes clearly presented in the findings? *See Results section.*
32.	Clarity of minor themes	Is there a description of diverse cases or discussion of minor themes? *See Results section.*
