# Supplementary material for: Online Safety When Considering Self-Harm and Suicide-Related Content: Qualitative Focus Group Study With Young People, Policy Makers, and Social Media Industry Professionals
Source: J Med Internet Res. 2025 Mar 10;27:e66321. doi: 10.2196/66321 (PMC11933773; doi:10.2196/66321)
Supplement: Multimedia Appendix 2 [file jmir_v27i1e66321_app2.doc]

**Semi-structured topic guide used to conduct the focus groups**

Part 1: Views regarding young people using social media to communicate about self-harm and suicide

1. What are your concerns when it comes to young people communicating online about their mental health, including self-harm and suicide?
2. Are there specific social media platforms or sites, or specific functions of these platforms, that concern you? (e.g., stories, no chat)
3. Do you think that social media platforms or sites could do more to keep young people safe? If so, what more do you think they should do?
4. Do you think that policymakers could do more to keep young people safe online? If so, what more do you think they should do?

Part 2: Thinking about Edition 2 of the (Resource name retracted)

Reminder to participants: When responding to these questions, please do not focus on feasibility issues and do not filter yourself.

1. What would you like to see included in the new guidelines that isn’t already there (i.e. in Edition 1)?
2. What other resources could be developed?
   - *Prompt: If we were to develop a training program, or provide information to specific groups, what sort of information would you like to see included and how could we best deliver that information?*
3. On a broader systems level, what else do you think could improve online safety for young people?
