# Supplementary material for: Online Safety When Considering Self-Harm and Suicide-Related Content: Qualitative Focus Group Study With Young People, Policy Makers, and Social Media Industry Professionals
Source: J Med Internet Res. 2025 Mar 10;27:e66321. doi: 10.2196/66321 (PMC11933773; doi:10.2196/66321)
Supplement: Multimedia Appendix 3 [file jmir_v27i1e66321_app3.doc]

**Thematic Framework**

| **Theme and Description** | **Sub-theme** | **Quote from each case** |
| --- | --- | --- |
| **1. Challenges and concerns**  This theme represents participants’ concerns associated with young people communicating online about self-harm and suicide (e.g., contagion, virality of content, exposure to harmful or graphic content). It also speaks to the primary challenge they identified in terms of differentiating harmful versus helpful content, and the fact that this may be different for each individual. Irrespective of these concerns, participants from all groups spoke about young people using, and continuing to use, social media to communicate about self-harm and suicide, citing a range of benefits (e.g., anonymity, lack of access to formal supports). Participant concerns were understood and expressed within the Australian social context, which features a constantly evolving public discourse that mostly associates social media with harm. | **1A.** Reasons for, and challenges related to, young people using social media to communicate about self-harm and suicide | **Young people:** *“I think another aspect of why young people felt so comfortable sharing and discussing content probably in the more negative way and an unsafe way was not only because they were anonymous, and what they could post would be deleted, but also with a cry for help. I find a lot of young people don't understand or have misunderstandings about reaching out for help; like talking to adults is terrifying and exposing that part is, for a young person as well who's probably just entered high school or learning about themselves, it's terrifying to reach out to someone who probably seems a bit more put together”*  **Policymakers:** “*These sites are quite invisible to the adult eye. It's very immersive for young people, but to us, it's all invisible and a lot of interactions are hidden*”  **Professionals from the social media industry:** *“[Using social media to reach out] It is basically, I'm going through something, and I just generally don't feel like I trust my school counsellor or my parent at home. I don't feel comfortable to reach out. I don't feel I'm at a stage here where I want to reach a hotline”* |
|  | **1B.** Reasoning with a deterministic narrative of harm | **Young people:** “*I've stumbled across places on the internet on social media and I'm like, oh my god, [it] hasn't really - really not doing great and making everyone else worse as a result*”  **Policymakers:** *“I just fundamentally think that being on social media is detriment to people’s mental health. So I’m sitting here struggling with, oh yeah, I think we need to do this. But how do we just get people off social media?”*  **Professionals from the social media industry:** *“...when is someone sitting in a park with their heel cut, when is that affirming that, I will heal and I'll get out into the sunshine again soon, and when is that damaging? It's very hard for us to know that. How are we going to have that sensible conversation if you can't have it in, ironically using this word, a safe space where it's not just going to be this sensationalised headline of, social media causes harm?”* |
| **2. Roles and Responsibilities**  This theme represents an awareness of different stakeholders who play a role in online safety and suicide prevention, however lines of responsibility were unclear. Individuals, governments, platforms and technology companies, traditional media, parents and schools were all mentioned by participants in this study as stakeholders who play a role in online safety and suicide prevention. However, it was not clear what their responsibilities were exactly, or where their responsibility ended. Due to this complexity, a sub-theme within the data speaks to the need to work together and establish better collaborations between all relevant parties. | **2A.** Who is responsible and where does responsibility start and stop? | **Young people:** “*I don't think that government policy can keep up with the evolution of social media and to the ways - or mental health, or mental health crises, or the ways young people communicate. By the time they pass whatever, who knows how we will be communicating? So that's not to say it's not their job, I just don't think that official policy might be the [route]”*  *"I don't think at that age we'd be able to handle all that responsibility because we are quite young…. I wouldn’t really view being educated as a responsibility. I'd find it almost like - I think - knowing more about it would make it less likely to happen"*  **Policymakers:** *“It is really tricky because it is so kind of dispersed. Both in terms of different, even departments in Australia that are responsible for different areas of online safety, but also, as you said, a lot of these organisations are international. So yeah, I think it kind of dilutes that responsibility a little bit”*  **Professionals from the social media industry:** *“One distinction I want to make really quickly though that I think is in part a challenge that comes through sometimes is that, we're not a medical company and we're not a healthcare company. While there's a lot that I feel that we may want to do, we are challenged by some of that other regulatory and obviously some red tape around where that line stops, and what we can do in terms of impacting [suicide and self injury content] across our platforms... That's not to say that we've not done a lot of great work and that we do continue to operate in the suicide and self-injury space, it's just I think there's significant limitations there because we just aren't a healthcare company”* |
|  | **2B.** The need for better collaborations | **Young people:** *“I feel like education - all of the stories that we've shared about that terrible period were about seeking help and not really having the resources or tools or vocabulary. So if you find a way to educate young people through the chatsafe guidelines in a more structured way, then I feel that would really alleviate a huge part of the problem”*  **Policymakers:** *"I just wanted to acknowledge the complexity and the rapidly changing environment that we're working in. I think it's an issue for all of us to collectively try and work with. I think it's something that’s quite difficult to get the balance right between what governments should legislate for and control, versus what is individual responsibility. How you find that right balance between trying to look after young people but also let them have their own space and make their own decisions. So I think it's just a highly complex area"*  **Professionals from the social media industry:** *“[it] is going to have to include a multi-pronged approach. That includes some level of, depending on age, parental, familial, government, industry; all of these different factors I think need to come together to really, I think uniformly protect people. In the absence of that, I think it's too easy to go one way or the other. To try to put this all on young people, to try to put this on social media or the government, it leaves out so many different other parts of this puzzle that when together, you're best placed to build that front to protect users”* |
| **3. Future Approaches and Potential Solutions**  This theme represents current and future applications of social media as a possible tool in suicide prevention. This theme reflects participants’ perspectives on the use of current safety tools and policies embedded within social media platforms, as well as their ideas for the future in utilising new technologies (e.g., AI). | **3A.** Acknowledging the limitations of current safety tools and policies | **Young people:** *“Personally, I don't have much faith in social media's reporting features. There have been times - like you see some really atrocious things online, or even hate speech; it doesn't need to be visual, and you're like, oh my gosh, I really - I can't believe I encountered that. That is such an awful, awful thing for you to say. You report it and then five days later it's like, it's been reviewed and it's been decided there was no issue here”*  **Policymakers:** "*It's very nuanced in terms of what rules you could hold companies to in terms of what content [they allow], because at the end of the day, they're a platform, not the organisation posting or not the thing posting on the internet. They are the platform in which people post content on…. It's like a car. The manufacturer of a vehicle isn't the driver. But if they don't ensure all of the necessary safety features and that causes injury and harm, that's another [issue]"*  **Professionals from the social media industry:** *“people would yell at me and be like, why can't you algorithmically detect self-harming content? I'd be like, the technology is not there yet. It doesn't mean it won't be, it's just not there yet. Then… people were being like, how dare you remove our content? It was like, whoa, whoa. This is a really important conversation to be having”* |
|  | **3B.** Scope for innovation and new ideas | **Young people:** *“They could be doing more... but more proactively in terms of, it seems like this post has content discussing suicide, click here for more information, and there's the chatsafe guidelines, or an iteration. It would definitely boost how social media is regarded”*  **Policymakers:** *“I definitely do think that having links and support, if - with the algorithm picking up [that certain] content - but also if the algorithm can pick up that kind of content, shouldn't there also be a few ways to hopefully stop that content, rather than just leaving it up and be like, here's some links though just in case you might click on it and have a look”*  **Professionals from the social media industry:** *“I think that my dream would be to have better help tooling available online for folk, so really having things like having a safety plan available or having a single session intervention that is culturally responsive and appropriate available for users to be able to opt into …. that’s evidence-based. I think that there is steps that the industry is taking towards getting there, I think that it will be interesting to see what happens in this space within the next five to 10 years”* |
